# Dimethyl (2*Z*)-2-[4-((1*Z*)-1-{2-[(2*Z*,5*Z*)-5-(2-meth­oxy-2-oxo­ethyl­idene)-4-oxo-3-phenyl-1,3-thia­zolidin-2-yl­idene]hydra­zin-1-yl­idene}eth­yl)anilino]but-2-ene­dio­ate

**DOI:** 10.1107/S1600536813032042

**Published:** 2013-11-30

**Authors:** Shaaban K. Mohamed, Mehmet Akkurt, Joel T. Mague, Alaa A. Hassan, Mustafa R. Albayati

**Affiliations:** a Chemistry and Environmental Division, Manchester Metropolitan University, Manchester M1 5GD, England; b Chemistry Department, Faculty of Science, Minia University, 61519 El-Minia, Egypt; c Department of Physics, Faculty of Sciences, Erciyes University, 38039 Kayseri, Turkey; d Department of Chemistry, Tulane University, New Orleans, LA 70118, USA; e Kirkuk University, College of Science, Department of Chemistry, Kirkuk, Iraq

## Abstract

The mol­ecule of the title compound, C_26_H_24_N_4_O_7_S, adopts a *trans* conformation about the central N—N bond, presumably to minimize steric between the substituents on these two atoms. An intra­molecular N—H⋯O hydrogen bond occurs. The phenyl ring is disordered over two sets of sites, with an occupancy ratio of 0.624 (8):0.376 (8). The azolidine ring is essentially planar [maximum deviation = 0.008 (5) Å] and makes a dihedral angle of 4.3 (2)° with the benzene ring and dihedral angles of 74.1 (3) and 69.1 (5)°, respectively, with the mean planes of the major and minor components of the disordered phenyl ring. The packing in the crystal is aided by the formation of several weak C—H⋯O and C—H⋯N inter­actions.

## Related literature

For the biological activity of thia­zolidinene-containing compounds, see: Chaudhari *et al.* (1975 ▶); Chaudhary *et al.* (1976 ▶); Babaoglu *et al.* (2003 ▶); Dwivedi *et al.* (1972 ▶); Parmar *et al.* (1972 ▶); Bondock *et al.* (2007 ▶); Vicini *et al.* (2008 ▶); Gududuru *et al.* (2004 ▶); Ottanà *et al.* (2005 ▶); Agrawal *et al.* (2000 ▶); Diurno *et al.* (1999 ▶); Omar *et al.* (2010 ▶); Vigorita *et al.* (2003 ▶); Rawal *et al.* (2005 ▶); Suzuki *et al.* (1999 ▶).
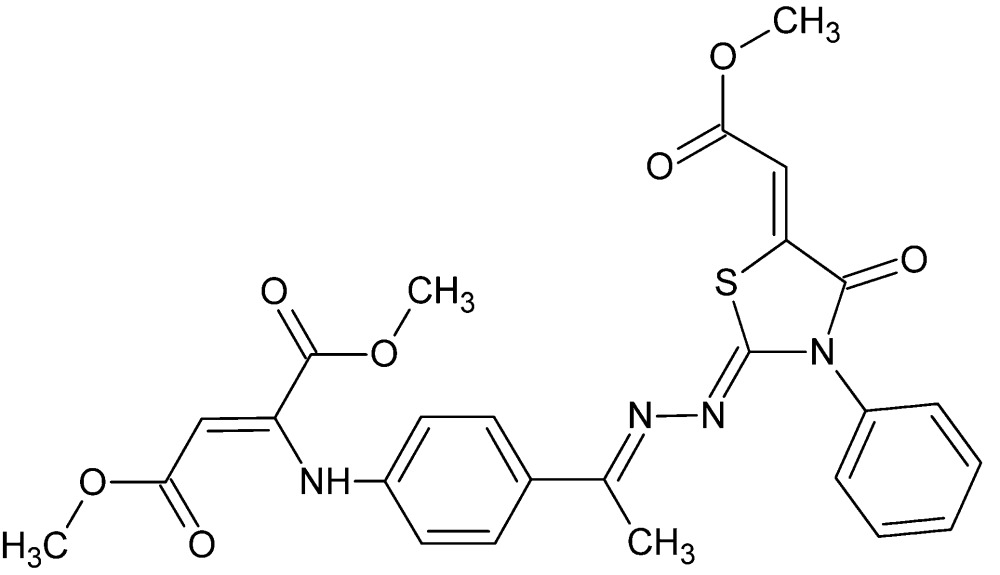


## Experimental

### 

#### Crystal data

C_26_H_24_N_4_O_7_S
*M* *_r_* = 536.56
Monoclinic, 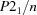

*a* = 15.7027 (6) Å
*b* = 4.8543 (2) Å
*c* = 33.5974 (13) Å
β = 92.539 (3)°
*V* = 2558.47 (17) Å^3^
*Z* = 4
Cu *K*α radiation
μ = 1.59 mm^−1^
*T* = 100 K
0.16 × 0.03 × 0.03 mm

#### Data collection

Bruker D8 VENTURE PHOTON 100 CMOS diffractometer
Absorption correction: multi-scan (*SADABS*; Bruker, 2013 ▶) *T*_min_ = 0.83, *T*_max_ = 0.95
11600 measured reflections
3655 independent reflections
2351 reflections with *I* > 2σ(*I*)
*R*_int_ = 0.106
θ_max_ = 59.1°

#### Refinement

*R*[*F* ^2^ > 2σ(*F* ^2^)] = 0.070
*wR*(*F* ^2^) = 0.156
*S* = 1.03
3655 reflections
339 parameters
6 restraints
H-atom parameters constrained
Δρ_max_ = 0.30 e Å^−3^
Δρ_min_ = −0.28 e Å^−3^

### 

Data collection: *APEX2* (Bruker, 2013 ▶); cell refinement: *SAINT* (Bruker, 2013 ▶); data reduction: *SAINT*; program(s) used to solve structure: *SHELXT* (Sheldrick, 2008 ▶); program(s) used to refine structure: *SHELXL2013* (Sheldrick, 2008 ▶); molecular graphics: *ORTEP-3 for Windows* (Farrugia, 2012 ▶); software used to prepare material for publication: *WinGX* (Farrugia, 2012 ▶) and *PLATON* (Spek, 2009 ▶).

## Supplementary Material

Crystal structure: contains datablock(s) global, I. DOI: 10.1107/S1600536813032042/sj5373sup1.cif

Structure factors: contains datablock(s) I. DOI: 10.1107/S1600536813032042/sj5373Isup2.hkl

Supplementary material file. DOI: 10.1107/S1600536813032042/sj5373Isup3.cml

Additional supplementary materials:  crystallographic information; 3D view; checkCIF report

## Figures and Tables

**Table 1:** Hydrogen-bond geometry (Å, °)

*D*—H⋯*A*	*D*—H	H⋯*A*	*D*⋯*A*	*D*—H⋯*A*
N4—H4⋯O6	0.88	2.01	2.705 (5)	135
C1—H1*B*⋯O4^i^	0.98	2.54	3.402 (8)	147
C3—H3⋯O3^ii^	0.95	2.37	3.191 (6)	145
C8*B*—H8*B*⋯N2^iii^	0.95	2.58	3.461 (8)	155
C12*B*—H12*B*⋯O3^iv^	0.95	2.31	3.216 (7)	159
C14—H14*A*⋯O6^v^	0.98	2.50	3.375 (6)	148

Symmetry codes: (i) 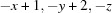; (ii) 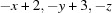; (iii) 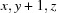; (iv) 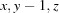; (v) 
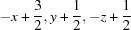
.

**Fig. 1.**
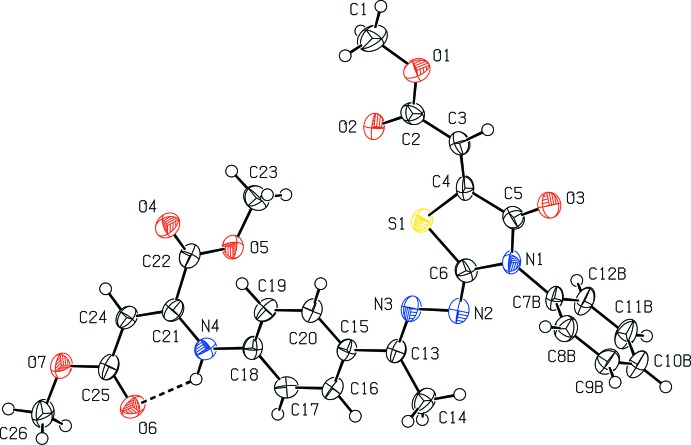
Perspective view of the title molecule showing the intramolecular hydrogen bond (dashed line). Displacement ellipsoids are drawn at the 50% probability level. For clarity only the major disorder component of the disordered phenyl ring is shown.

**Fig. 2.**
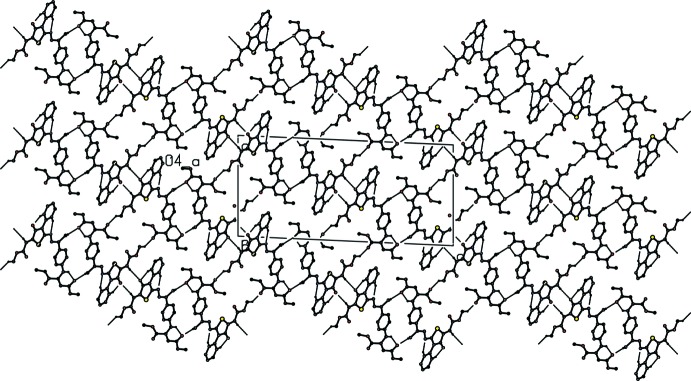
Packing of the title molecule view down b showing the intermolecular hydrogen bonds as dashed lines.

## supplementary crystallographic information

### 1. Comment

The 4-thiazolidinone ring system is a core structure in various synthetic compounds and an important scaffold known to be associated with several biological activities such as hypnotic activity (Chaudhari *et al.*, 1975; Chaudhary *et al.*, 1976), anti-tubercular (Babaoglu *et al.*, 2003), anti-convulsant (Dwivedi *et al.*, 1972; Parmar *et al.*, 1972), anti-bacterial (Bondock *et al.*, 2007; Vicini *et al.*, 2008), anti-cancer (Gududuru *et al.*, 2004; Ottanà *et al.*, 2005), anti-histaminic (Agrawal *et al.*, 2000; Diurno *et al.*, 1999), anti-fungal (Omar *et al.*, 2010), anti-inflammatory (Vigorita *et al.*, 2003), anti-viral (Rawal *et al.*, 2005) properties and cardiovascular effects (Suzuki *et al.*, 1999). Based on such findings and further to our studies on synthesis of a series of thiazolidinones, we report herein the synthesis and crystal structure of the title compound.

The title molecule (I), (Fig. 1), adopts a *trans* conformation about the central N2—N3 bond, presumably to minimize contact between the substituents on these two atoms. The conformation about the N4—C21 bond is determined by the presence of an intramolecular N4—H1···O6 hydrogen bond (Fig. 1 and Table 1). The azolidine ring (S1/N1/C4–C6) is essentially planar [maximum deviation = 0.008 (5) Å for C5] and makes a dihedral angle of 4.3 (2)° with the benzene ring (C15–C20) and dihedral angles of 74.1 (3) and 69.1 (5)°, respectively, with the mean planes of the major and minor components (C7B–C12B and C7A–C12A) of the disordered phenyl ring.

In the crystal, the molecular packing is stabilized by the several weak C—H···O and C—H···N intermolecular interactions (Fig. 2 and Table 1).

### 2. Experimental

A mixture of 283 mg (1 mmol) (2E)-2-[1-(4-aminophenyl)ethylidene]-*N*-phenylhydrazinecarbothioamide and 284 mg (2 mmol) dimethyl but-2-ynedioate in 50 ml of ethanol was refluxed and monitored by TLC until completion of the reaction. The excess solvent was evaporated under vacuum and the solid obtained was recrystallized from ethanol to afford clear yellow crystals (*M*.p. 443–445 K) of X-ray quality.

IR: 3420 (NH), 1742, 1717, 1665 (CO), 1599(Ar—C=C). ^1^H-NMR (CDCl_3_) δ=2.24 (s,3*H*,CH_3_), 3.76(s,3*H*,OCH_3_), 3.78(s,3*H*,OCH_3_), 3.89(s,3*H*,OCH_3_), 6.86 (s,1*H*,vinyl-CH), 6.88(s,1*H*,vinyl-CH), 7.43–7.48 (m, 4H,Ar—H), 7.52–7.57 (m,3*H*,Ar—H), 7.83–7.87 (m,2*H*,Ar—H), 9.75 (br,1*H*,NH). ^13^C-NMR (CDCl_3_) δ=14.84 (CH_3_), 51.36, 52.52, 53.03 (OCH_3_), 116.02 (vinyl-CH), 119.61 (vinyl-CH), 127.44, 128.02, 128.91, 129.09, 129.93 (Ar—CH), 132.73, 141.97 (Ar—C), 142.15 (=C—NH), 146.88 (acyclic C=N), 158.27 (thiazole-C2), 164.03 (cyclic C=O), 164.61, 166.62, 169.66 (ester C=O).

### 3. Refinement

H atoms attached to carbon were positioned geometrically while that attached to nitrogen was placed in a location derived from a difference map. All were allowed to ride on their parent atoms with N—H = 0.91 Å, C—H = 0.95 and 0.98 Å, with *U*_iso_(H) = 1.5 *U*_iso_(C) for CH_3_ H atoms and *U*_iso_(H) = 1.2 *U*_iso_(C,*N*) for the other H atoms. The phenyl ring attached to the N atom of the thiazolidine ring is disordered over two sites in a 0.624 (8):0.376 (8) ratio. The two orientations were refined as rigid groups with AFIX 66 and EADP instructions. The small proportion of reflections observed is a result of the rather poor quality of the very thin crystal specimens.

### Crystal data

**Table:**

C_26_H_24_N_4_O_7_S	*F*(000) = 1120
*M**_r_* = 536.56	*D*_x_ = 1.393 Mg m^−^^3^
Monoclinic, *P*2_1_/*n*	Cu *K*α radiation, λ = 1.54178 Å
Hall symbol: -P 2yn	Cell parameters from 9855 reflections
*a* = 15.7027 (6) Å	θ = 3.1–61.2°
*b* = 4.8543 (2) Å	µ = 1.59 mm^−^^1^
*c* = 33.5974 (13) Å	*T* = 100 K
β = 92.539 (3)°	Column, yellow
*V* = 2558.47 (17) Å^3^	0.16 × 0.03 × 0.03 mm
*Z* = 4	

### Data collection

**Table:**

Bruker D8 VENTURE PHOTON 100 CMOS diffractometer	3655 independent reflections
Radiation source: INCOATEC IµS micro–focus source	2351 reflections with *I* > 2σ(*I*)
Mirror monochromator	*R*_int_ = 0.106
Detector resolution: 10.4167 pixels mm^-1^	θ_max_ = 59.1°, θ_min_ = 2.6°
ω scans	*h* = −17→17
Absorption correction: multi-scan (*SADABS*; Bruker, 2013)	*k* = −5→5
*T*_min_ = 0.83, *T*_max_ = 0.95	*l* = −37→31
11600 measured reflections	

### Refinement

**Table:**

Refinement on *F*^2^	6 restraints
Least-squares matrix: full	Hydrogen site location: inferred from neighbouring sites
*R*[*F*^2^ > 2σ(*F*^2^)] = 0.070	H-atom parameters constrained
*wR*(*F*^2^) = 0.156	W = 1/[Σ^2^(*F*_o_^2^) + (0.0309*P*)^2^ + 6.4905*P*] where *P* = (*F*_o_^2^ + 2*F*_c_^2^)/3
*S* = 1.03	(Δ/σ)_max_ < 0.001
3655 reflections	Δρ_max_ = 0.30 e Å^−^^3^
339 parameters	Δρ_min_ = −0.28 e Å^−^^3^

### Special details

**Table:**

Geometry. Bond distances, angles *etc*. have been calculated using the rounded fractional coordinates. All su's are estimated from the variances of the (full) variance-covariance matrix. The cell e.s.d.'s are taken into account in the estimation of distances, angles and torsion angles
Refinement. Refinement on *F*^2^ for ALL reflections except those flagged by the user for potential systematic errors. Weighted *R*-factors *wR* and all goodnesses of fit *S* are based on *F*^2^, conventional *R*-factors *R* are based on *F*, with *F* set to zero for negative *F*^2^. The observed criterion of *F*^2^ > σ(*F*^2^) is used only for calculating -*R*-factor-obs *etc*. and is not relevant to the choice of reflections for refinement. *R*-factors based on *F*^2^ are statistically about twice as large as those based on *F*, and *R*-factors based on ALL data will be even larger.

### Fractional atomic coordinates and isotropic or equivalent isotropic displacement parameters (Å^2^)

**Table:**

	*x*	*y*	*z*	*U*_iso_*/*U*_eq_	Occ. (<1)
S1	0.82910 (8)	0.9838 (3)	0.07318 (4)	0.0352 (4)	
O1	0.7365 (2)	1.5472 (9)	−0.02761 (10)	0.0509 (14)	
O2	0.7018 (2)	1.2215 (9)	0.01654 (11)	0.0503 (14)	
O3	1.0163 (2)	1.4497 (8)	0.04611 (11)	0.0500 (14)	
O4	0.4439 (2)	−0.0248 (8)	0.10592 (10)	0.0485 (14)	
O5	0.5638 (2)	−0.2773 (7)	0.10342 (10)	0.0383 (12)	
O6	0.4864 (2)	−0.5316 (7)	0.24001 (10)	0.0420 (12)	
O7	0.3549 (2)	−0.6713 (8)	0.22011 (10)	0.0474 (14)	
N1	0.9907 (2)	1.0985 (9)	0.08935 (12)	0.0346 (14)	
N2	0.9367 (3)	0.7543 (9)	0.12948 (12)	0.0370 (16)	
N3	0.8626 (3)	0.5940 (8)	0.13394 (13)	0.0353 (14)	
N4	0.5687 (2)	−0.2105 (8)	0.18830 (12)	0.0331 (14)	
C1	0.6494 (4)	1.5545 (15)	−0.04284 (18)	0.066 (3)	
C2	0.7544 (3)	1.3729 (12)	0.00233 (15)	0.0383 (17)	
C3	0.8436 (3)	1.3888 (11)	0.01578 (15)	0.0372 (17)	
C4	0.8772 (3)	1.2395 (11)	0.04549 (15)	0.0338 (17)	
C5	0.9686 (3)	1.2793 (12)	0.05910 (15)	0.0382 (17)	
C6	0.9255 (3)	0.9273 (11)	0.10114 (15)	0.0366 (17)	
C7B	1.0773 (3)	1.0893 (13)	0.1080 (2)	0.0320 (17)	0.624 (8)
C8B	1.1061 (4)	1.2977 (13)	0.1336 (2)	0.044 (3)	0.624 (8)
C9B	1.1888 (4)	1.2897 (13)	0.1501 (2)	0.046 (3)	0.624 (8)
C10B	1.2426 (3)	1.0733 (14)	0.1411 (2)	0.045 (2)	0.624 (8)
C11B	1.2137 (4)	0.8649 (12)	0.1155 (2)	0.051 (3)	0.624 (8)
C12B	1.1311 (4)	0.8729 (12)	0.0990 (2)	0.045 (3)	0.624 (8)
C13	0.8620 (3)	0.4521 (10)	0.16606 (14)	0.0300 (17)	
C14	0.9306 (3)	0.4598 (11)	0.19870 (15)	0.0426 (17)	
C15	0.7858 (3)	0.2779 (10)	0.17131 (13)	0.0287 (17)	
C16	0.7861 (3)	0.0648 (10)	0.19881 (14)	0.0312 (17)	
C17	0.7147 (3)	−0.0942 (10)	0.20385 (14)	0.0327 (17)	
C18	0.6403 (3)	−0.0423 (10)	0.18172 (14)	0.0300 (17)	
C19	0.6384 (3)	0.1794 (10)	0.15518 (14)	0.0336 (17)	
C20	0.7097 (3)	0.3316 (10)	0.14958 (14)	0.0317 (17)	
C21	0.4985 (3)	−0.2571 (10)	0.16418 (14)	0.0324 (17)	
C22	0.4970 (3)	−0.1693 (11)	0.12144 (15)	0.0354 (17)	
C23	0.5758 (4)	−0.1888 (14)	0.06307 (15)	0.055 (2)	
C24	0.4309 (3)	−0.4101 (10)	0.17585 (15)	0.0345 (17)	
C25	0.4288 (3)	−0.5354 (11)	0.21454 (16)	0.0364 (17)	
C26	0.3515 (4)	−0.8178 (14)	0.25707 (17)	0.058 (2)	
C12A	1.0914 (6)	1.165 (3)	0.1430 (3)	0.045 (3)	0.376 (8)
C9A	1.2206 (5)	0.972 (3)	0.0964 (3)	0.046 (3)	0.376 (8)
C7A	1.0723 (5)	1.075 (2)	0.1044 (3)	0.0320 (17)	0.376 (8)
C8A	1.1370 (6)	0.978 (3)	0.0811 (3)	0.044 (3)	0.376 (8)
C10A	1.2397 (5)	1.063 (3)	0.1350 (3)	0.045 (2)	0.376 (8)
C11A	1.1750 (7)	1.159 (3)	0.1583 (3)	0.051 (3)	0.376 (8)
H1B	0.64500	1.67420	−0.06630	0.0980*	
H1A	0.63100	1.36790	−0.05030	0.0980*	
H11B	1.25050	0.71690	0.10940	0.0610*	0.624 (8)
H1C	0.61300	1.62650	−0.02230	0.0980*	
H3	0.87960	1.51280	0.00250	0.0450*	
H4	0.56980	−0.29720	0.21130	0.0400*	
H8B	1.06940	1.44570	0.13970	0.0530*	0.624 (8)
H9B	1.20860	1.43210	0.16760	0.0550*	0.624 (8)
H10B	1.29910	1.06780	0.15240	0.0540*	0.624 (8)
H19	0.58670	0.22460	0.14090	0.0400*	
H20	0.70770	0.47660	0.13060	0.0380*	
H23A	0.52380	−0.22530	0.04670	0.0830*	
H23B	0.62350	−0.29000	0.05220	0.0830*	
H23C	0.58810	0.00910	0.06280	0.0830*	
H24	0.38350	−0.43460	0.15770	0.0410*	
H26A	0.39650	−0.95760	0.25860	0.0870*	
H26B	0.29580	−0.90740	0.25860	0.0870*	
H26C	0.35980	−0.68840	0.27930	0.0870*	
H12B	1.11130	0.73040	0.08150	0.0540*	0.624 (8)
H14A	0.94080	0.27310	0.20900	0.0640*	
H14B	0.91260	0.57890	0.22030	0.0640*	
H14C	0.98330	0.53230	0.18810	0.0640*	
H16	0.83660	0.02780	0.21450	0.0370*	
H17	0.71670	−0.24050	0.22270	0.0390*	
H8A	1.12400	0.91620	0.05470	0.0530*	0.376 (8)
H9A	1.26480	0.90630	0.08050	0.0550*	0.376 (8)
H10A	1.29680	1.05890	0.14550	0.0540*	0.376 (8)
H11A	1.18800	1.22130	0.18470	0.0610*	0.376 (8)
H12A	1.04720	1.23120	0.15890	0.0540*	0.376 (8)

### Atomic displacement parameters (Å^2^)

**Table:**

	*U*^11^	*U*^22^	*U*^33^	*U*^12^	*U*^13^	*U*^23^
S1	0.0316 (7)	0.0325 (7)	0.0414 (7)	−0.0036 (6)	−0.0005 (6)	0.0001 (6)
O1	0.045 (2)	0.059 (3)	0.048 (2)	0.007 (2)	−0.0057 (18)	0.011 (2)
O2	0.032 (2)	0.067 (3)	0.052 (2)	−0.008 (2)	0.0031 (18)	0.006 (2)
O3	0.042 (2)	0.058 (3)	0.050 (2)	−0.015 (2)	0.0031 (18)	0.009 (2)
O4	0.037 (2)	0.054 (3)	0.054 (2)	0.010 (2)	−0.0040 (18)	0.014 (2)
O5	0.032 (2)	0.042 (2)	0.041 (2)	0.0025 (18)	0.0033 (16)	−0.0009 (17)
O6	0.040 (2)	0.041 (2)	0.044 (2)	−0.0105 (19)	−0.0094 (18)	0.0036 (18)
O7	0.034 (2)	0.056 (3)	0.052 (2)	−0.0129 (19)	−0.0018 (18)	0.014 (2)
N1	0.025 (2)	0.039 (3)	0.040 (2)	−0.006 (2)	0.0037 (19)	0.000 (2)
N2	0.030 (2)	0.034 (3)	0.047 (3)	−0.001 (2)	0.003 (2)	−0.002 (2)
N3	0.029 (2)	0.028 (2)	0.049 (3)	−0.002 (2)	0.003 (2)	−0.001 (2)
N4	0.034 (3)	0.028 (2)	0.037 (2)	−0.004 (2)	−0.002 (2)	0.0022 (19)
C1	0.053 (4)	0.083 (5)	0.060 (4)	0.020 (4)	−0.011 (3)	0.008 (4)
C2	0.042 (3)	0.041 (3)	0.032 (3)	0.003 (3)	0.004 (3)	−0.004 (3)
C3	0.035 (3)	0.039 (3)	0.038 (3)	−0.006 (3)	0.006 (3)	0.004 (3)
C4	0.025 (3)	0.036 (3)	0.041 (3)	−0.006 (2)	0.007 (2)	−0.004 (3)
C5	0.037 (3)	0.043 (3)	0.035 (3)	−0.005 (3)	0.005 (2)	0.000 (3)
C6	0.037 (3)	0.035 (3)	0.038 (3)	−0.002 (3)	0.004 (2)	−0.002 (3)
C7B	0.021 (3)	0.033 (3)	0.042 (3)	−0.004 (2)	0.002 (2)	−0.005 (3)
C8B	0.037 (5)	0.047 (5)	0.048 (5)	0.005 (4)	0.002 (4)	−0.009 (5)
C9B	0.037 (5)	0.056 (6)	0.045 (5)	0.007 (5)	−0.003 (4)	−0.018 (5)
C10B	0.024 (3)	0.051 (4)	0.059 (4)	0.002 (3)	−0.008 (3)	−0.007 (3)
C11B	0.028 (5)	0.063 (7)	0.062 (6)	0.008 (5)	−0.002 (4)	−0.026 (5)
C12B	0.023 (5)	0.054 (6)	0.058 (6)	−0.001 (4)	−0.004 (4)	−0.015 (5)
C13	0.026 (3)	0.022 (3)	0.042 (3)	0.006 (2)	0.001 (2)	−0.002 (2)
C14	0.034 (3)	0.037 (3)	0.056 (3)	−0.001 (3)	−0.008 (3)	0.010 (3)
C15	0.027 (3)	0.025 (3)	0.034 (3)	−0.001 (2)	0.000 (2)	−0.005 (2)
C16	0.028 (3)	0.025 (3)	0.040 (3)	0.001 (2)	−0.004 (2)	−0.002 (2)
C17	0.040 (3)	0.024 (3)	0.034 (3)	0.002 (2)	−0.001 (2)	0.000 (2)
C18	0.028 (3)	0.024 (3)	0.038 (3)	−0.006 (2)	0.002 (2)	−0.006 (2)
C19	0.032 (3)	0.023 (3)	0.045 (3)	0.004 (2)	−0.007 (2)	0.000 (2)
C20	0.028 (3)	0.025 (3)	0.042 (3)	0.000 (2)	0.000 (2)	−0.004 (2)
C21	0.029 (3)	0.028 (3)	0.040 (3)	0.007 (2)	−0.002 (2)	−0.001 (2)
C22	0.029 (3)	0.031 (3)	0.046 (3)	0.000 (3)	−0.001 (3)	0.001 (3)
C23	0.049 (4)	0.075 (5)	0.042 (3)	0.009 (3)	0.009 (3)	0.006 (3)
C24	0.029 (3)	0.032 (3)	0.042 (3)	0.004 (2)	−0.004 (2)	0.002 (2)
C25	0.027 (3)	0.030 (3)	0.052 (3)	−0.002 (3)	−0.002 (3)	−0.002 (3)
C26	0.038 (4)	0.071 (4)	0.064 (4)	−0.019 (3)	0.000 (3)	0.021 (4)
C12A	0.023 (5)	0.054 (6)	0.058 (6)	−0.001 (4)	−0.004 (4)	−0.015 (5)
C9A	0.037 (5)	0.056 (6)	0.045 (5)	0.007 (5)	−0.003 (4)	−0.018 (5)
C7A	0.021 (3)	0.033 (3)	0.042 (3)	−0.004 (2)	0.002 (2)	−0.005 (3)
C8A	0.037 (5)	0.047 (5)	0.048 (5)	0.005 (4)	0.002 (4)	−0.009 (5)
C10A	0.024 (3)	0.051 (4)	0.059 (4)	0.002 (3)	−0.008 (3)	−0.007 (3)
C11A	0.028 (5)	0.063 (7)	0.062 (6)	0.008 (5)	−0.002 (4)	−0.026 (5)

### Geometric parameters (Å, º)

**Table:**

S1—C4	1.743 (5)	C13—C14	1.503 (7)
S1—C6	1.767 (5)	C15—C20	1.397 (7)
O1—C1	1.439 (7)	C15—C16	1.387 (7)
O1—C2	1.335 (7)	C16—C17	1.378 (7)
O2—C2	1.219 (6)	C17—C18	1.380 (7)
O3—C5	1.210 (6)	C18—C19	1.397 (7)
O4—C22	1.192 (6)	C19—C20	1.361 (7)
O5—C22	1.341 (6)	C21—C22	1.497 (7)
O5—C23	1.442 (6)	C21—C24	1.367 (7)
O6—C25	1.217 (6)	C24—C25	1.437 (7)
O7—C25	1.355 (6)	C1—H1A	0.9800
O7—C26	1.434 (7)	C1—H1B	0.9800
N1—C5	1.376 (7)	C1—H1C	0.9800
N1—C6	1.390 (6)	C3—H3	0.9500
N1—C7B	1.473 (6)	C8A—H8A	0.9500
N1—C7A	1.361 (9)	C8B—H8B	0.9500
N2—N3	1.413 (6)	C9A—H9A	0.9500
N2—C6	1.276 (7)	C9B—H9B	0.9500
N3—C13	1.281 (6)	C10A—H10A	0.9500
N4—C18	1.415 (6)	C10B—H10B	0.9500
N4—C21	1.358 (6)	C11A—H11A	0.9500
N4—H4	0.8800	C11B—H11B	0.9500
C2—C3	1.455 (7)	C12A—H12A	0.9500
C3—C4	1.324 (7)	C12B—H12B	0.9500
C4—C5	1.499 (7)	C14—H14A	0.9800
C7A—C12A	1.389 (15)	C14—H14B	0.9800
C7A—C8A	1.392 (14)	C14—H14C	0.9800
C7B—C8B	1.390 (9)	C16—H16	0.9500
C7B—C12B	1.390 (8)	C17—H17	0.9500
C8A—C9A	1.389 (13)	C19—H19	0.9500
C8B—C9B	1.390 (9)	C20—H20	0.9500
C9A—C10A	1.390 (15)	C23—H23B	0.9800
C9B—C10B	1.390 (9)	C23—H23C	0.9800
C10A—C11A	1.390 (15)	C23—H23A	0.9800
C10B—C11B	1.391 (9)	C24—H24	0.9500
C11A—C12A	1.389 (14)	C26—H26B	0.9800
C11B—C12B	1.388 (9)	C26—H26C	0.9800
C13—C15	1.482 (7)	C26—H26A	0.9800
			
C4—S1—C6	90.8 (2)	O4—C22—O5	125.4 (5)
C1—O1—C2	116.7 (4)	C21—C24—C25	122.6 (4)
C22—O5—C23	116.4 (4)	O6—C25—O7	121.6 (5)
C25—O7—C26	115.2 (4)	O6—C25—C24	125.7 (5)
C5—N1—C6	115.3 (4)	O7—C25—C24	112.7 (4)
C5—N1—C7B	122.1 (4)	O1—C1—H1A	109.00
C5—N1—C7A	122.0 (5)	O1—C1—H1B	109.00
C6—N1—C7B	122.6 (4)	O1—C1—H1C	109.00
C6—N1—C7A	122.5 (5)	H1A—C1—H1B	109.00
N3—N2—C6	110.8 (4)	H1A—C1—H1C	110.00
N2—N3—C13	115.0 (4)	H1B—C1—H1C	109.00
C18—N4—C21	129.2 (4)	C2—C3—H3	118.00
C21—N4—H4	115.00	C4—C3—H3	118.00
C18—N4—H4	115.00	C7A—C8A—H8A	120.00
O1—C2—C3	111.6 (4)	C9A—C8A—H8A	120.00
O2—C2—C3	124.8 (5)	C9B—C8B—H8B	120.00
O1—C2—O2	123.6 (4)	C7B—C8B—H8B	120.00
C2—C3—C4	123.5 (5)	C10A—C9A—H9A	120.00
S1—C4—C5	111.1 (4)	C8A—C9A—H9A	120.00
S1—C4—C3	128.6 (4)	C8B—C9B—H9B	120.00
C3—C4—C5	120.2 (5)	C10B—C9B—H9B	120.00
N1—C5—C4	110.5 (4)	C11A—C10A—H10A	120.00
O3—C5—C4	125.3 (5)	C9A—C10A—H10A	120.00
O3—C5—N1	124.2 (4)	C11B—C10B—H10B	120.00
S1—C6—N1	112.3 (4)	C9B—C10B—H10B	120.00
S1—C6—N2	125.8 (4)	C10A—C11A—H11A	120.00
N1—C6—N2	121.8 (4)	C12A—C11A—H11A	120.00
N1—C7A—C8A	121.2 (8)	C10B—C11B—H11B	120.00
C8A—C7A—C12A	119.9 (8)	C12B—C11B—H11B	120.00
N1—C7A—C12A	118.8 (8)	C7A—C12A—H12A	120.00
N1—C7B—C12B	119.3 (5)	C11A—C12A—H12A	120.00
C8B—C7B—C12B	120.0 (5)	C11B—C12B—H12B	120.00
N1—C7B—C8B	120.7 (5)	C7B—C12B—H12B	120.00
C7A—C8A—C9A	120.0 (10)	C13—C14—H14A	110.00
C7B—C8B—C9B	119.9 (6)	C13—C14—H14C	109.00
C8A—C9A—C10A	120.0 (9)	H14A—C14—H14B	109.00
C8B—C9B—C10B	120.0 (6)	C13—C14—H14B	110.00
C9A—C10A—C11A	119.9 (8)	H14B—C14—H14C	109.00
C9B—C10B—C11B	120.0 (5)	H14A—C14—H14C	109.00
C10A—C11A—C12A	120.1 (10)	C15—C16—H16	119.00
C10B—C11B—C12B	120.0 (5)	C17—C16—H16	119.00
C7A—C12A—C11A	120.0 (9)	C18—C17—H17	120.00
C7B—C12B—C11B	120.0 (6)	C16—C17—H17	120.00
N3—C13—C15	116.3 (4)	C18—C19—H19	120.00
C14—C13—C15	118.9 (4)	C20—C19—H19	120.00
N3—C13—C14	124.7 (4)	C15—C20—H20	119.00
C13—C15—C20	120.5 (4)	C19—C20—H20	119.00
C13—C15—C16	121.7 (4)	O5—C23—H23B	110.00
C16—C15—C20	117.7 (4)	O5—C23—H23C	110.00
C15—C16—C17	121.4 (4)	H23A—C23—H23C	109.00
C16—C17—C18	120.4 (4)	H23B—C23—H23C	110.00
C17—C18—C19	118.6 (4)	H23A—C23—H23B	109.00
N4—C18—C17	118.0 (4)	O5—C23—H23A	109.00
N4—C18—C19	123.3 (4)	C25—C24—H24	119.00
C18—C19—C20	120.8 (4)	C21—C24—H24	119.00
C15—C20—C19	121.1 (4)	O7—C26—H26C	109.00
N4—C21—C24	122.7 (4)	O7—C26—H26B	109.00
N4—C21—C22	120.2 (4)	H26B—C26—H26C	109.00
C22—C21—C24	116.8 (4)	H26A—C26—H26B	109.00
O5—C22—C21	110.0 (4)	H26A—C26—H26C	109.00
O4—C22—C21	124.6 (4)	O7—C26—H26A	110.00
			
C6—S1—C4—C3	178.8 (5)	S1—C4—C5—O3	−177.2 (5)
C6—S1—C4—C5	−0.7 (4)	C3—C4—C5—N1	−178.3 (5)
C4—S1—C6—N1	0.0 (4)	C3—C4—C5—O3	3.2 (8)
C4—S1—C6—N2	179.2 (5)	S1—C4—C5—N1	1.3 (5)
C1—O1—C2—C3	−178.6 (5)	N1—C7B—C12B—C11B	178.3 (5)
C1—O1—C2—O2	1.0 (8)	N1—C7B—C8B—C9B	−178.3 (6)
C23—O5—C22—C21	−174.0 (4)	C8B—C7B—C12B—C11B	−0.1 (10)
C23—O5—C22—O4	6.1 (7)	C12B—C7B—C8B—C9B	0.2 (10)
C26—O7—C25—C24	175.6 (5)	C7B—C8B—C9B—C10B	−0.2 (10)
C26—O7—C25—O6	−2.6 (7)	C8B—C9B—C10B—C11B	0.1 (10)
C5—N1—C6—N2	−178.4 (5)	C9B—C10B—C11B—C12B	−0.1 (10)
C7B—N1—C5—C4	179.1 (5)	C10B—C11B—C12B—C7B	0.1 (10)
C5—N1—C7B—C12B	−105.5 (7)	N3—C13—C15—C16	163.1 (5)
C6—N1—C5—O3	177.2 (5)	C14—C13—C15—C16	−19.2 (7)
C7B—N1—C5—O3	−2.4 (8)	C14—C13—C15—C20	158.1 (4)
C6—N1—C5—C4	−1.4 (6)	N3—C13—C15—C20	−19.5 (7)
C5—N1—C6—S1	0.8 (6)	C13—C15—C16—C17	179.2 (4)
C5—N1—C7B—C8B	72.9 (7)	C20—C15—C16—C17	1.8 (7)
C6—N1—C7B—C8B	−106.6 (7)	C16—C15—C20—C19	−0.1 (7)
C7B—N1—C6—N2	1.2 (8)	C13—C15—C20—C19	−177.6 (4)
C7B—N1—C6—S1	−179.6 (4)	C15—C16—C17—C18	−0.7 (7)
C6—N1—C7B—C12B	74.9 (7)	C16—C17—C18—C19	−2.0 (7)
N3—N2—C6—N1	−176.3 (4)	C16—C17—C18—N4	−179.5 (4)
N3—N2—C6—S1	4.6 (6)	C17—C18—C19—C20	3.7 (7)
C6—N2—N3—C13	−167.4 (5)	N4—C18—C19—C20	−179.0 (4)
N2—N3—C13—C15	−179.0 (4)	C18—C19—C20—C15	−2.6 (7)
N2—N3—C13—C14	3.5 (7)	C24—C21—C22—O4	61.8 (7)
C18—N4—C21—C24	−175.0 (5)	N4—C21—C22—O5	54.8 (6)
C21—N4—C18—C19	23.3 (8)	C22—C21—C24—C25	172.4 (5)
C18—N4—C21—C22	12.5 (7)	N4—C21—C22—O4	−125.3 (5)
C21—N4—C18—C17	−159.3 (5)	N4—C21—C24—C25	−0.3 (8)
O2—C2—C3—C4	−0.5 (9)	C24—C21—C22—O5	−118.2 (5)
O1—C2—C3—C4	179.1 (5)	C21—C24—C25—O7	178.4 (5)
C2—C3—C4—C5	−176.5 (5)	C21—C24—C25—O6	−3.5 (8)
C2—C3—C4—S1	4.0 (8)		

### Hydrogen-bond geometry (Å, º)

**Table:**

*D*—H···*A*	*D*—H	H···*A*	*D*···*A*	*D*—H···*A*
N4—H4···O6	0.88	2.01	2.705 (5)	135
C1—H1*B*···O4^i^	0.98	2.54	3.402 (8)	147
C3—H3···O3^ii^	0.95	2.37	3.191 (6)	145
C8*B*—H8*B*···N2^iii^	0.95	2.58	3.461 (8)	155
C12*B*—H12*B*···O3^iv^	0.95	2.31	3.216 (7)	159
C14—H14*A*···O6^v^	0.98	2.50	3.375 (6)	148
C14—H14*C*···N2	0.98	2.33	2.735 (7)	104

Symmetry codes: (i) −*x*+1, −*y*+2, −*z*; (ii) −*x*+2, −*y*+3, −*z*; (iii) *x*, *y*+1, *z*; (iv) *x*, *y*−1, *z*; (v) −*x*+3/2, *y*+1/2, −*z*+1/2.
